# Developing ClerkCast: An Emergency Medicine Clerkship Needs Assessment Project

**DOI:** 10.7759/cureus.7459

**Published:** 2020-03-29

**Authors:** Ben Forestell, Lauren Beals, Ajay Shah, Teresa M Chan

**Affiliations:** 1 Emergency Medicine, McMaster University, Hamilton, CAN; 2 Emergency Medicine, Mcmaster University, Hamilton, CAN; 3 Orthopaedics, Michael G. DeGroote School of Medicine, McMaster University, Hamilton, CAN

**Keywords:** clerkship, podcast, needs assessment, emergency medicine, free open access medical education

## Abstract

Introduction and Objectives: For Canadian medical students completing their emergency medicine (EM) clerkship rotation, developing an approach to undifferentiated patients can be difficult. Open educational resources (OERs) are a convenient solution, but faculty authored materials may not meet students’ needs. There is a lack of EM OERs that deconstruct these undifferentiated EM presentations for medical students. The objective of this study was to identify EM topics poorly understood by medical students to inform a novel Free Open Access Medical Education podcast curriculum for approaching undifferentiated EM patients for medical students.

Methods: An online survey-based needs assessment was distributed to key stakeholders through direct email, social media, and the blog CanadiEM. The survey included 32 EM topics graded on a five-point Likert scale according to how much participants believe medical students require further teaching.

Results: Over six weeks, a total of 74 participants completed the needs assessment survey, and 58 participants met the criteria for inclusion into our study: medical students (n=23) and EM educators (inclusive of resident physicians (n=19), and staff EM physicians (n=16)). A number of presentations (n=23) were prioritized by both students and EM educators to be of the greatest need for medical students. No presentations identified as high priority by students were not also identified as high priority by EM educators.

Conclusions: The greatest mean topic scores in both EM educators and medical student responses included critical care and acute medicine topics. Of the 32 topics in the survey, 23 topics were determined to be high priority for the development of future online educational resources. Analysis of free-text responses revealed nine topics not previously listed in our survey. Our findings will be used to inform the development of our new open access podcast and can be useful for developing medical student curricula in EM.

## Introduction

Free Open Access Medical education (FOAM) is a movement within medicine where educators create content for free distribution online [1]. The accessibility of FOAM online educational resources (OERs) has made it an essential tool in the emergency department (ED), with 99% of Canadian emergency medicine (EM) residents using FOAM for their EM learning [2].

EM is a mandatory core clerkship rotation in Canadian medical schools [3]. On their rotations, students are tasked with caring for patients with a wide range of chief complaints, but may lack the clinical experience to formulate an appropriate differential for these cases. Teachers often also make cognitive jumps when explaining diagnostic reasoning [4,5]. However, the majority of EM OERs feature content rarely applicable to junior learners, often glossing over the foundational knowledge to target practicing EM physicians [6].

We sought to identify knowledge gaps in EM core rotations by asking both students and educators which topics they believed required further teaching for medical students, with the goal of using our results to inform a novel FOAM podcast.

## Materials and methods

Materials

Our study received an exemption from our institutional review board (the Hamilton Integrated Research Ethics Board). Per the Tri-Council policy statement in Canada, program development is exempt.

The needs assessment survey was created on Google Forms (Mountainview, CA, USA) and was available over six weeks in the spring of 2019. The survey topics included the 24 cardinal EM presentations from the EM textbook Rosen’s Emergency Medicine and an additional eight topics of importance as determined by the consensus amongst the authors [7].

On a five-point Likert scale, respondents were asked to determine to which degree each of the selected EM topics required further education for clinical clerks as compared to rotational objectives, with selections ranging from “not at all” to “very high degree”. Respondents could also suggest other EM topics through a free-text response box. Respondents were asked basic demographic questions such as professional role and affiliated medical school. Medical students were also asked about completion of their EM clerkship rotation.

The link to the form was distributed via email to all 17 Canadian medical school EM interest groups, EM chief residents, and EM clerkship directors. These medical schools each represent a university, and encompass multiple affiliated academic and community hospitals. The form was also hosted on the Canadian FOAM website CanadiEM.org and shared through the CanadiEM social media accounts (Twitter, Facebook).

We included responses from emergency physicians, EM residents, and medical students who had completed at least one clerkship rotation in EM at the time of completing the survey. Responses from outside of Canada were excluded.

Analysis

Demographics were analyzed using Excel (Microsoft Corp., Seattle, WA). Likert scale responses were converted to integer values from 1 to 5, and then organized into two groups: EM educators (including resident physicians in EM training programs) and medical students. Mean topic scores greater than 3 for either group were determined to be high priority topics relevant for the development of future online educational resources. Median topic scores were compared using the Mann-Whitney signed-rank test under the null hypothesis that there would be no difference between educators and students.

Free-text responses were independently analyzed for frequently mentioned topics. Two study authors (B.F. and L.B.) separately reviewed the qualitative comments for new topics not previously found within our survey. A third author (T.C.) reviewed the list of topics to ensure trustworthiness of the analysis.

## Results

Demographics

Over six weeks, 74 participants completed the needs assessment survey, with 58 responses meeting the inclusion criteria. Responses included EM staff physicians (n=10, 17.2%), EM clerkship directors (n=4, 6.9%, a response rate of 4/17, 23.5%), EM program directors (n=2, 3.5%), resident physicians (n=19, 32.8%), and medical students (n=23, 39.7%). The response rate from EM clerkship directors was all medical students had completed their core EM clerkship rotation, and the majority of medical students had additional exposure to EM through electives in EM (n=14, 60.8%).

Responses were geographically clustered within Canada, with the majority of responses from just two medical schools (53.4%, n=31), and with the majority of the 17 Canadian medical schools with under three responses (64.7%, n=11).

Quantitative analysis

Median EM educator topic scores were significantly higher than medical students’ using the Wilcoxon signed-rank test (p<0.0005), suggesting that EM educators perceive greater need for further student education on EM topics than students do themselves.

**Figure 1:**
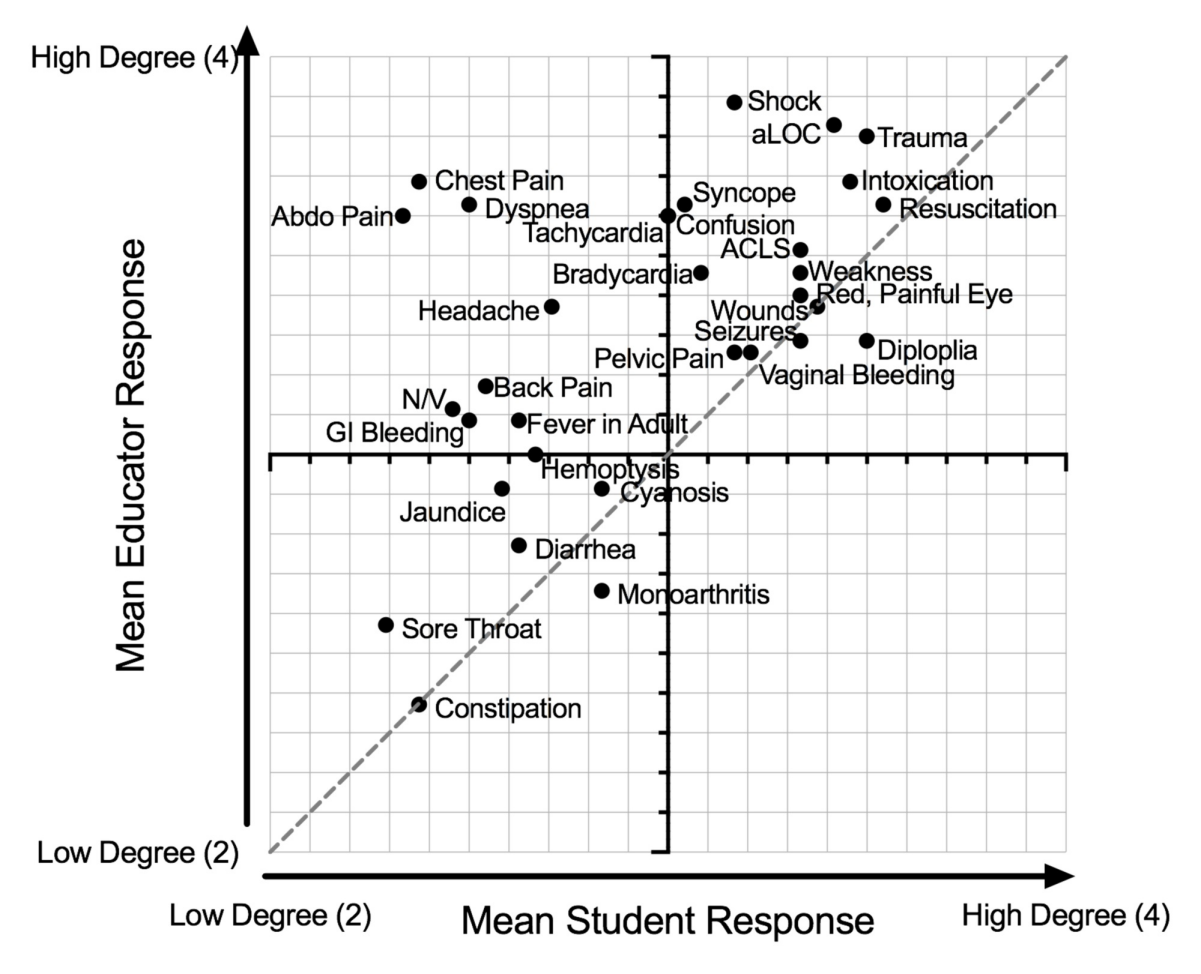
Prioritization of EM topics by medical students and EM educators. Mean topic scores of EM topics, plotted medical students vs EM educators. EM, emergency medicine.

Plotted mean topic scores of groups highlighted shared perceptions of educational needs for medical students in EM (Figure 1). The greatest mean topic scores in both EM educators and medical student responses included critical care and acute medicine topics. Of the 32 topics in the survey, 23 topics were determined to be high priority for the development of future online educational resources.

Qualitative analysis

**Figure 2:**
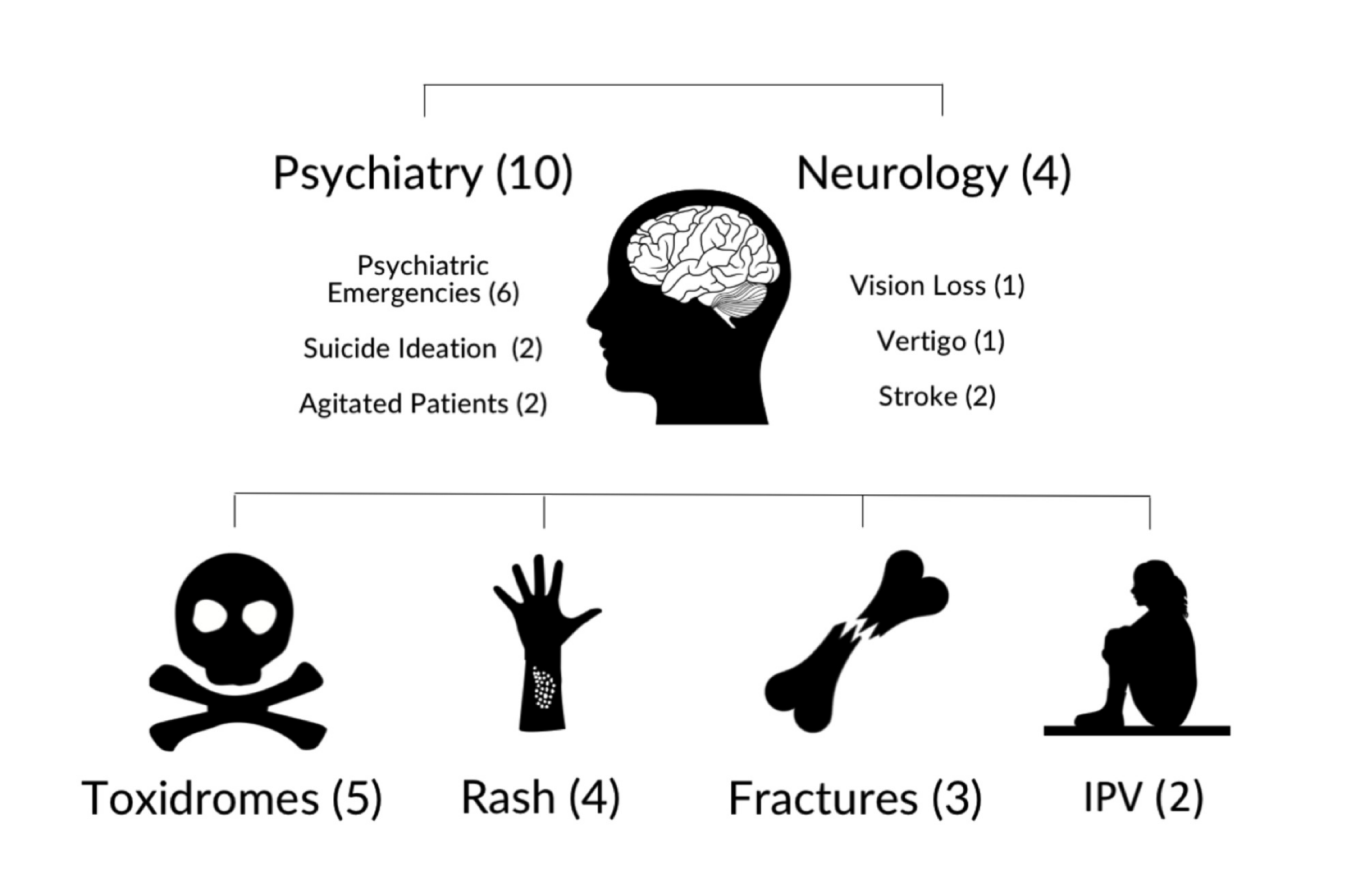
Additional EM topics as suggested by EM educators and trainees in free-text responses. EM, emergency medicine; IPV, intimate partner violence; number in brackets = frequency of free-text response.

Analysis of free-text responses revealed nine topics not previously listed in our survey. These are listed in Figure 2. The topics were as follows: toxidromes, rash, fractures, intimate-partner violence, psychiatry (psychiatric emergencies, suicidal ideation, and agitated patients), and neurology (vision loss, vertigo, and stroke). 

## Discussion

In the online needs assessment, proposed topics were stratified according to the need for further education based on educator and medical student responses. Summative data will be used to create online resources for Canadian medical students for their EM rotation, with high priority topics being translated into podcast episodes and blog posts. Our highlighted topics closely follow the Medical Expert (presenting problem) competencies for EM clerkship in Canada, and therefore our podcast will be an appropriate complement to Canadian EM clerkship curricula [4]. In doing so, we aim to harness the power of the cognitive apprenticeship framework to help clerks understand what their attendings might be thinking as they approach cases in the ED [8].

Generally, topics that students identify as high priority are also deemed a high priority by the educator group, but some differences were noted. For instance, educators believe that medical students require education on topics such as chest pain, abdominal pain, and dyspnea (cardinal EM presentations) more than medical students viewed themselves. This finding may represent the Dunning-Kruger effect, wherein medical students perceive themselves to be more competent than their current knowledge base in core topics [9]. While these common presentations may be frequently seen by a medical student on their EM rotation, these are presentations for which education persists into residency and beyond [10].

Unsurprisingly, both EM educator and medical student responses highlighted the need for further education on critical care topics. The EM core clerkship rotation is an important introduction to resuscitation and trauma, and medical students have little to no introduction to these topics in other clerkship rotations [4]. Critical care cases are high acuity, but low frequency, and may benefit from further education to provide students with a framework prior to clinical exposures, perhaps through the integration of more simulation.

Limitations

Our data may be subject to responder bias due to the small sample size of both EM educators and medical student responses. In particular, there was a small sample of medical student responses, which may reflect that although EM interest group leadership at each school was contacted with the survey, it is possible they did not forward the survey on to other students within their local networks. Furthermore, the disproportionately increased number of responses from two of the 17 Canadian medical schools may limit the validity of these results outside of these medical schools.

Due to selection of medical students with EM experience, the perceived needs of medical student responses also reflect the needs of more experienced students rather than those entering their core EM rotation initially. Investigations to determine EM topics requiring further education from medical students who have not completed their clerkship rotation may be difficult insofar as these preclinical students may not be aware of their own deficiencies in EM knowledge.

## Conclusions

Through our survey of Canadian stakeholder groups (medical students, EM residents, EM faculty members, and EM clerkship directors), we were able to identify the key educational needs of medical students during their EM core rotation. Our findings will be used to inform the development of our new open access podcast, “ClerkCast”. The podcast will aim to address important gaps in Canadian medical student knowledge and improve clinical performance on their EM core rotation. This list of topics may also be useful for developing other medical student curricula in EM.
